# Cause inhabituelle d´une occlusion colique, sténose anale post-radique: à propos d´un cas et revue de la littérature Post-radiation anal stenosis as an unusual cause of colic occlusion: case study and literature review

**DOI:** 10.11604/pamj.2020.37.195.23625

**Published:** 2020-10-29

**Authors:** Wilson Bizimana, Raïssa Kaukone, Hounayda Jerguigue, Rachida Latib, Youssef Omor

**Affiliations:** 1 Service de Radiologie, Institut National d´Oncologie, Centre Hospitalier Universitaire Ibn Sina, Rabat, Maroc

**Keywords:** Occlusion colique, radiothérapie, sténose anale, imagerie, Colonic obstruction, radiotherapy, anal stenosis, imaging

## Abstract

Résumé

L´occlusion radique est une complication grave de l´entéropathie radique. Elle survient chez les patients sous radiothérapie suivis pour cancer gynécologique ou du rectum. Sa prise en charge nécessite une attention particulière pour améliorer la survie de ces patients. A partir d´un cas ayant été opéré pour cancer du rectum et qui a présenté une occlusion sur sténose serrée post-radique du canal anal et de la partie distale du sigmoïde, nous allons décrire la physiopathologie de cette entité rare et illustrer la valeur de l´imagerie dans la prise en charge de cette pathologie.

English abstract

Post-radiation occlusion is a serious complication of radiation enteropathy. It occurs in patients undergoing radiotherapy for gynecologic or rectal cancer. Accurate management is essential to improve patients' survival. We here report the case of a patient undergoing surgery for rectal cancer. He had post-radiation stricture due to tight stenosis of the anal canal and of the distal end of the sigmoid colon. This study describes the pathophysiology of this rare entity and highlights the role of imaging tests in the management of this disorder.

## Introduction

La radiothérapie constitue une modalité importante de traitement pour le cancer du rectum, du col, de l´utérus, de la vessie, de la prostate et des testicules [1]. Cinquante pourcent (50%) des patients reçoivent la radiothérapie au cours du traitement des principaux cancers [2]. Les effets nocifs de la radiothérapie peuvent survenir précocement ou tardivement même après la résolution du problème pour lequel il était indiqué. Dans le tractus gastrointestinal, les lésions peuvent concerner tous les segments [2]. La sténose anale et sigmoïdienne post-radique n´est pas commentée dans la littérature à notre connaissance. Nous rapportons un cas d´occlusion sur sténose post-radique survenue après 5 ans et un accent sera mis sur la physiopathologie et le rôle de l´imagerie pour confirmer le diagnostic.

## Patient et observation

**Figure 1:**
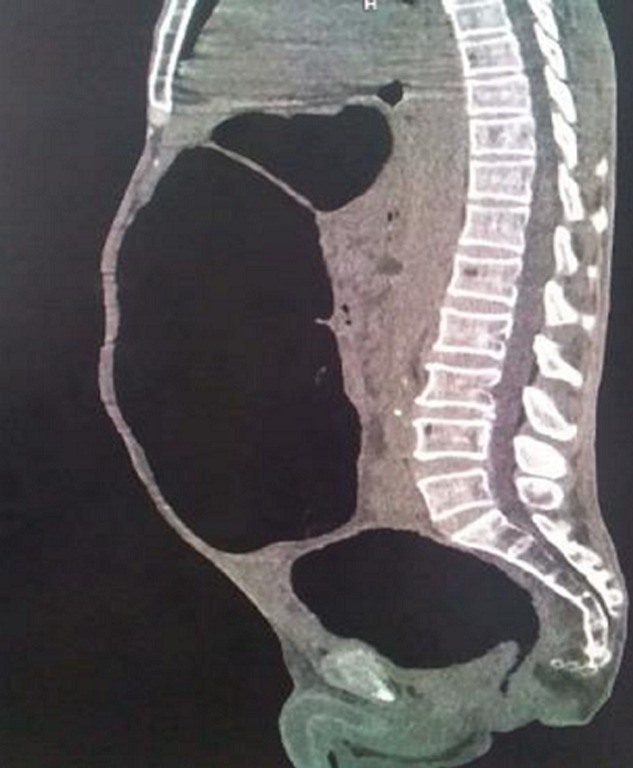
TDM abdominopelvienne sans injection, coupe sagittale: épaississement sténosant du canal anal et du sigmoïde avec distension colique diffuse en amont

**Figure 2:**
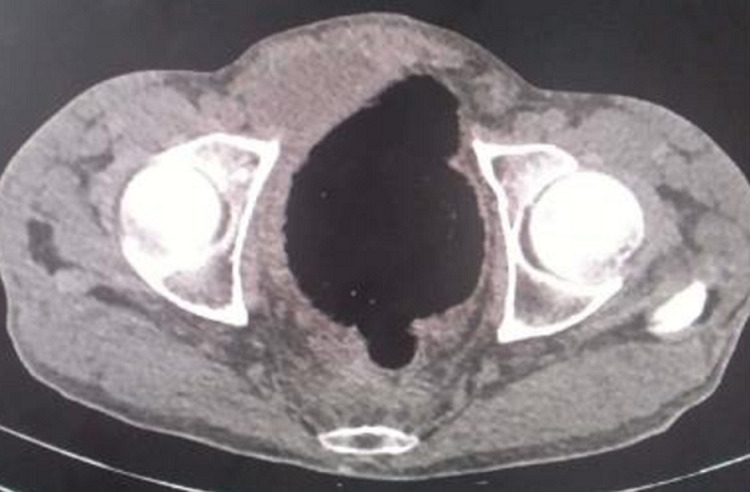
TDM abdominopelvienne sans injection, coupe coronale, notant la sténose anale

**Figure 3:**
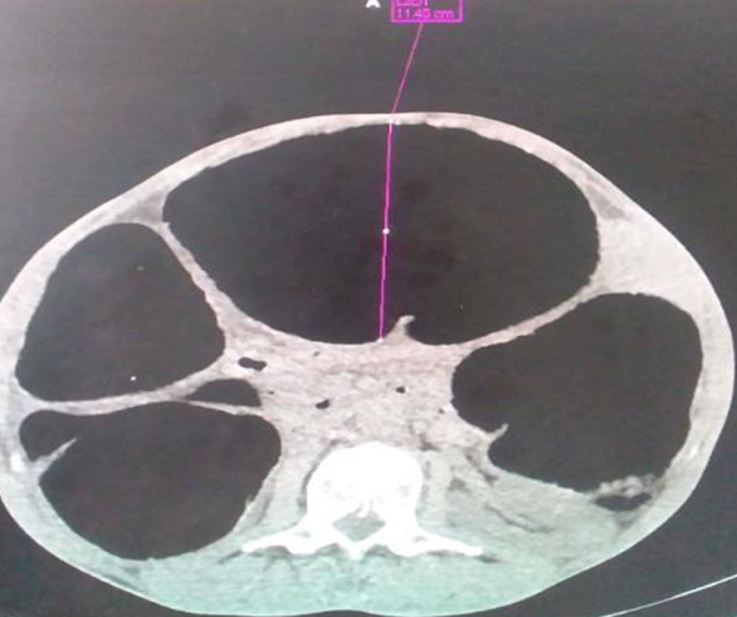
TDM abdominopelvienne sans injection, coupe axiale montrant la sténose du canal anal avec distension digestive gazeuse en amont

**Figure 4:**
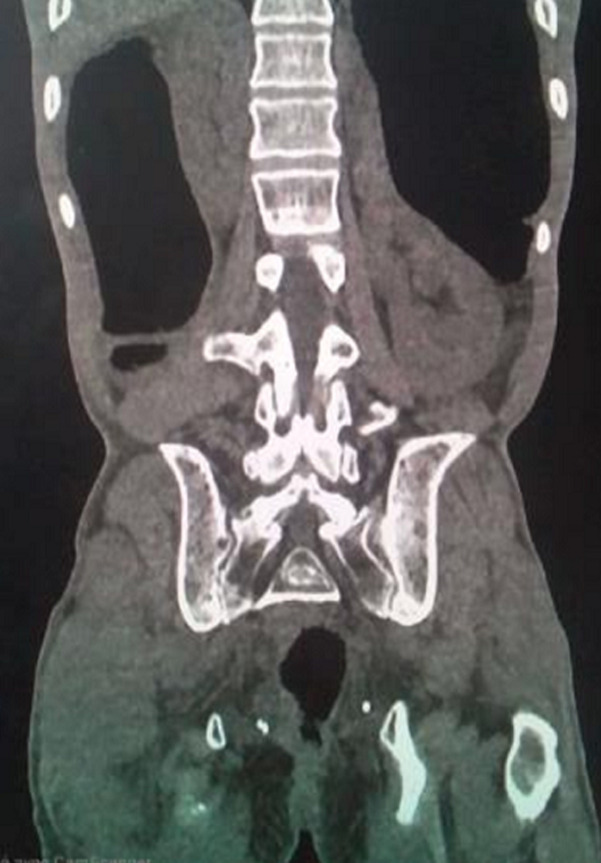
TDM abdominopelvienne sans injection, coupe axiale, notant la distension colique diffuse de contenu aérique

**Figure 5:**
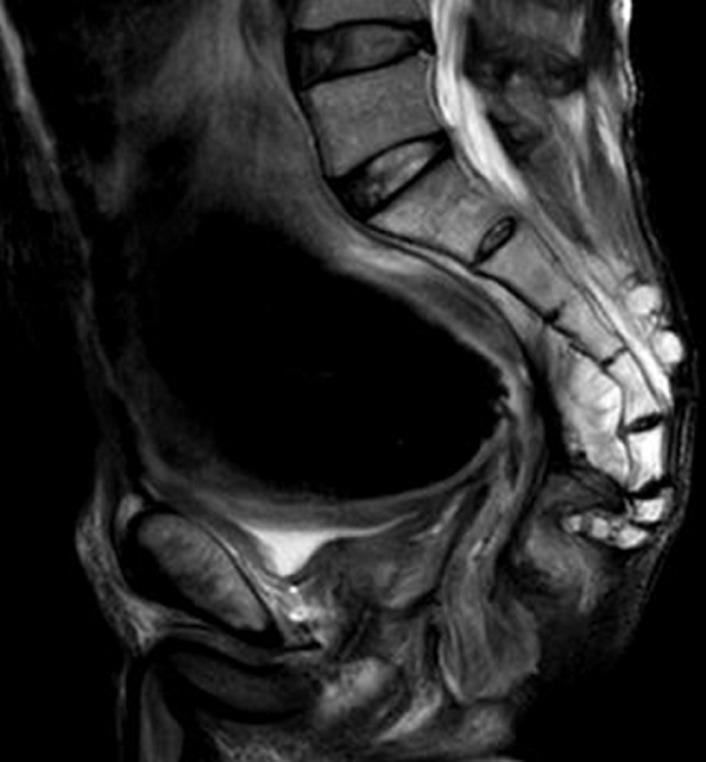
IRM pelvienne, coupe sagittale séquence pondérée en T2: épaississement sténosant en signal intermédiaire avec distension colique en amont et effet compressif sur la vessie

**Figure 6:**
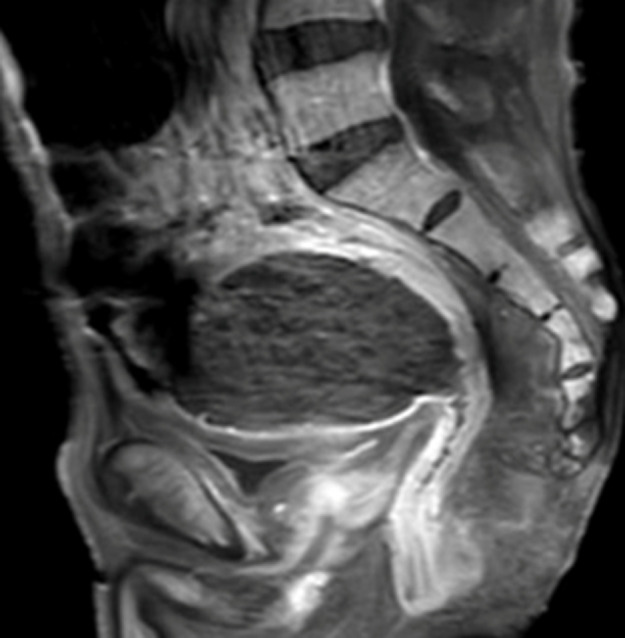
IRM pelvienne, coupe sagittale, séquence pondérée en T1 avec injection au Gadolinium: rehaussement modéré de l´épaississement sténosant du canal anal et du bas sigmoïde

Il s´agit d´un patient de sexe masculin, âgé de 70 ans, suivi pour tumeur du bas rectum. Il a bénéficié en 2015, d´une amputation abdomino-pelvienne (AAP) + colostomie périnéale pseudo-continente (CPC) et puis traité par radiothérapie et chimiothérapie postopératoire. Après 5 ans, le patient est réadmis pour un syndrome occlusif. Un scanner abdominal demandé en urgence a montré un épaississement régulier circonférentiel et sténosant du canal anal et de la partie distale du côlon abouchée à l´anus comme le montrent les coupes sagittales coronale et axiale du scanner (Figure 1, Figure 2 et Figure 3). En plus, cet épaississement entrainait, en amont, une importante distension colique diffuse de contenu aérique mesurée à 13 cm (Figure 4). Pour évaluer la nature de l´épaississement et trancher entre une CPC trop serrée, une récidive ou un deuxième cancer qui devrait être confirmé par une preuve histologique et considérant la lourdeur de la deuxième chirurgie chez un patient déjà affaibli par l´âge et par sa maladie, un complément d´IRM abdominopelvienne a été fait pour faire la part des choses. Nous avons réalisé les séquences pondérées en T1, T2, diffusion, et T1 FATSAT avec injection de Gadolinium. L´IRM a noté un épaississement sténosant du canal anal et de la partie distale du côlon abouché à l´anus associé à une importante distension et stase digestive en amont de la sténose qui exerçait une compression sur la vessie (Figure 5). Cet épaississement présentait un rehaussement modéré après injection du Gadolinium avec la CPC paraissant imperméable (Figure 6) sans restriction de la diffusion (absence d´hypersignal sur la séquence de la diffusion. Enfin, le diagnostic d´occlusion post-radique a été retenu. Le patient a bénéficié d´une colostomie droite avec dilatation colo-anale par bougie d´Hégar.

## Discussion

L´entéropathie radique est un désordre fonctionnel du tractus intestinal qui survient pendant ou après la radiothérapie au niveau de l´abdomen, du pelvis ou du rectum [3]. La radiothérapie est utilisée comme traitement indépendant, ou traitement adjuvent dans les cancers gynécologiques et comme traitement néoadjuvant dans le cancer rectal. Les lésions intestinales obstructives radio-induites représentent entre 0,8 et 17%. Son étiopathogénie complexe ne dépend pas seulement de surdosage ou de la technique utilisée mais peut survenir à tout moment postopératoire chez le patient ayant subi une chirurgie et sous radiothérapie néoadjuvante [4, 5].

**Physiopathologie:** des facteurs favorisants une toxicité radique sont connus. La littérature distingue les facteurs liés au patient et les facteurs liés à la radiothérapie. Le premier groupe renferme l´obésité, l´HTA, la consommation tabagique, l´artériopathie, le diabète et l´antécédent de maladie inflammatoire grêlique et colique, antécédent de chirurgie abdominale et de péritonite. Le deuxième groupe regroupe une dose >45 Gy avec une dose Journalière > 2 Gy, la radiothérapie postopératoire étant plus toxique que celle préopératoire [5, 6]. Une chirurgie abdominale ou pelvienne antérieure a été associée à un risque accru de développer des obstructions chez les patients qui reçoivent > 50 Gy [7]. La toxicité aigüe débute après quelques jours à 6 mois de la radiothérapie tandis que la toxicité chronique s´observe entre 18 mois et 30ans après la radiothérapie [4]. L´irradiation génère des radicaux libres qui sont responsables de la rupture de l´ADN simple ou double brin, l´atteinte de la membrane cytoplasmique et création de l´apoptose [6]. Après l´inflammation, la radiothérapie va induire une fibrose responsable de l´obstruction et obstacle à l´écoulement du contenu digestif pouvant indiquer une urgence médicochirurgicale [8]. En plus de l´obstruction, l´irradiation induit tardivement une ulcération, une perforation, un saignement et une fistule [7, 8]. L´occlusion secondaire à une sténose post-radique est une forme grave de la complication d´entérite radique [4]. Notre cas a eu une atteinte du canal anal sur toute sa longueur et du sigmoïde abouché à l´anus (Figure 5).

**Diagnostic:** la symptomatologie est superposable à celle des occlusions en général et regroupe les nausées, les vomissements, les douleurs abdominales, la distension abdominale et l´arrêt des matières et des gaz [6]. L´imagerie prend une place incontournable pour confirmer le diagnostic alors qu´avant la confirmation se faisait par l´histologie après laparotomie ou biopsie endoscopique. La tendance actuelle repose sur l´imagerie surtout avec l´évolution de l´IRM, ce qui a modifié aussi le traitement. Elle permet un diagnostic positif, étiologique, de siège, l´étendue, et le degré en précisant les éventuelles complications (perforation, fistule, abcès ou collections) [8].

**La tomodensitométrie (TDM):** permet le diagnostic positif de l´occlusion. Le taux de diagnostic de l´obstruction intestinale est estimé à 73 et 95% [8]. Le scanner a un taux de sensibilité de 63%, spécificité de 78% avec une précision de 66% [9]. Grace au scanner, on individualise la zone de disparité de calibre en amont de laquelle siège une distension digestive supérieur à 50mm pour le colon et supérieur à 25mm pour la grêle avec un épaississement circonférentiel régulier sténosant et mesure son étendue. En cas de radiothérapie chez un patient opéré pour cancer digestif, le challenge du scanner est la non spécificité de trancher entre récidive tumorale, second cancer radio-induit ou toxicité radique. La TDM avec opacification à la gastrografine peut montrer, dans les suites de la radiothérapie pelvienne, les segments lésés et aider à prévenir l´évolution vers l´obstruction [3].

**L´imagerie par résonance magnétique (IRM):** est le gold standard du diagnostic. C´est une imagerie qui n´expose plus le patient aux rayons X et préserve au malade de subir un geste lourd, facilite le diagnostic précoce et oriente le traitement. Un système de 1,5 Tesla est fréquemment utilisé. L´administration d´antipéristaltiques ou d´antispasmodiques améliore la qualité des images. Les séquences morphologiques T1 avant et après injection de chélate de Gadolinium et la séquence T2 sont requises. La séquence de la diffusion qui confirme le diagnostic est fortement recommandée dans cette indication et participe à éliminer les diagnostics différentiels [8]. Elle note l´absence de restriction de la diffusion. Selon la haute autorité de santé, la radiographie d´abdomen sans préparation utilisée dans les études antérieures, a peu d´intérêts actuellement [2].

**Diagnostics différentiels:** l´imagerie contribue à éliminer les principaux diagnostics différentiels à savoir l´iléus reflexe, l´ischémie mésentérique, les métastases, la récidive tumorale ou le second cancer radio-induit, la sclérose ou fibrose postopératoire [9, 10].

**Traitement:** l´entérite radique peut être prise en charge de façon conservatrice par la corticothérapie ou d´autres agents antiinflammatoires ou l´application endoscopique de formol dans les intestins [1]. En cas d´entéropathie chronique compliquée d´obstruction, la chirurgie par laparotomie ou laparoscopie est indiquée pour éviter une nécrose progressive, un séjour hospitalier prolongé et voire une mortalité [1]. Elle consiste à faire une résection du segment atteint et faire une anastomose. Néanmoins, on note une augmentation du pourcentage de réintervention en cas d´occlusion post-radique chez les patients opérés qui est estimé entre 34 à 60% [4]. Pour pallier à ce risque et augmenter la survie des patients, on opte pour une dilatation endoscopique par ballonnet associée à une injection intra lésionnelle de corticothérapie et faire une colostomie simple de décharge. Le taux de réussite pour cette technique avoisine 97% avec un risque < 3% [6]. La surveillance avec une imagerie de ces patients et la correction des désordres nutritionnels est un préalable pour prévenir ces complications sévères [6].

**Pronostic:** la morbidité postopératoire est de 30% alors que la mortalité due à l´occlusion radique oscille entre 10% et 33% [10]. La chirurgie est compliquée du fait de la fibrose étendue et des adhérences, pour ce, elle doit être évitée dans la mesure du possible.

## Conclusion

La sténose anale post-radique est une complication rare. Le clinicien et le radiologue doivent y penser en cas d´occlusion digestive chez le patient sous radiothérapie. L´IRM joue un rôle majeur pour confirmer le diagnostic et éliminer une cause maligne. Son traitement privilégie une dilatation endoscopique avec injection de corticoïdes pour prévenir la morbi-mortalité qui n´est pas rare dans cette population.
